# Application of a Quantitative PCR to Investigate the Distribution and Dynamics of Two Morphologically Similar Species, *Karenia mikimotoi* and *K. papilionacea* (Dinophyceae) in Korean Coastal Waters

**DOI:** 10.3390/toxins15070469

**Published:** 2023-07-20

**Authors:** Sunju Kim, Minji Cho, Jiae Yoo, Bum Soo Park

**Affiliations:** 1 Major of Oceanography, Division of Earth Environmental System Science, Pukyong National University, Busan 48513, Republic of Korea; 761aststar@gmail.com (M.C.); jiae502@gmail.com (J.Y.); 2 Department of Life Science, College of Natural Sciences, Hanyang University, Seoul 04763, Republic of Korea; 3 Research Institute for Convergence of Basic Science, Hanyang University, Seoul 04763, Republic of Korea; 4 Hanyang Institute of Bioscience and Biotechnology, Hanyang University, Seoul 04763, Republic of Korea; 5 Hanyang Institute of Advanced BioConvergence, Hanyang University, Seoul 04763, Republic of Korea

**Keywords:** harmful algal bloom species, toxic dinoflagellate, *Karenia* species, quantitative real-time PCR, field application

## Abstract

Species of the marine dinoflagellate genus *Karenia* are known to produce various potent biotoxins and can form noxious blooms that cause mass mortalities of fish and shellfish. To date, harmful blooms of the species *K. mikimotoi* have been reported in Korea, but *K. papilionacea* was recently recorded off the southern coast of Korea. Here, we developed a quantitative real-time PCR (qRT-PCR) assay with specific primer pairs for the accurate detection and quantification of these two similar-looking unarmored species, *K. mikimotoi* and *K. papilionacea,* and investigated their distribution and dynamics in Korean coastal waters. Overall, *K. papilionacea* had not only a wider distribution, but also higher cell abundances (15–2553 cells L^−1^) than *K. mikimotoi* (3–122 cells L^−1^) in surface waters. Of 18 sampling sites, the two *Karenia* species were found to coexist at two sites. During monitoring at a fixed station (S5), *K. papilionacea* was generally predominant over *K. mikimotoi*; however, the two species exhibited similar dynamics and occasionally co-occurred. Both *Karenia* species showed similar physiological responses to temperature and salinity, requiring similar conditions for optimum growth. These results suggest that blooms of the two species may co-occur and induce a synergistic adverse effect on marine environments.

## 1. Introduction

Several species of the marine unarmored dinoflagellate genus *Karenia* are known to produce various potent toxins (i.e., brevetoxins, gymnocins, gymnodimines, and unidentified ichthyotoxins) and can cause harmful algal blooms (HABs) in coastal and oceanic temperate waters. For instance, *Karenia brevis* produces neurotoxic brevetoxins. Noxious blooms of this species have been documented almost annually in the Gulf coasts of Florida, Texas, and Mexico, and in the Atlantic coast of Florida, leading to mass mortalities of fish, birds, and marine mammals and posing a risk to human health via neurotoxic shellfish poisoning (NSP) [1,2,3,4]. The cytotoxic species *K. mikimotoi* has a worldwide distribution and produces toxic and hemolytic compounds, including gymnocins and glycolipids that cause fish gill damage; although, the mechanism of toxicity remains unknown [5,6,7]. HABs of this species have been linked to serious damage to fisheries, particularly in Japan and Norway [8,9,10]. Other *Karenia* species are also known to generate ichthyotoxic metabolites, including neurotoxic gymnodimine in *K. selliformis* [11] and *brevisulcenals* toxins (KBTs) and brevisulcatic acids (BSX) in *K. brevisulcata* [12].

Often, *Karenia* toxigenic blooms involve more than one species. For example, HABs dominated by *K. brevis* often contain significant numbers of other species, such as *K. mikimotoi* and *K. papilionacea* [13]. Blooms of several *Karenia* species lead to the presence of multiple toxins. In New Zealand, during the 1992–1993 biotoxin event, multiple biotoxins (e.g., brevetoxins, gymnodimine, and domoic acid) were detected in shellfish and later toxins were reported to originate from several toxic phytoplankton species [6,14,15]. Numerous *Karenia* species were involved in this event, all initially identified as *K. brevis* but later reclassified as several new species [10]. Toxic blooms by multiple *Karenia* species make it more difficult to assess the risk to humans and environments posed by toxigenic species, which may also present synergistic toxic effects [10]. 

Unarmored *Karenia* species can be difficult to distinguish based on morphology under a light microscope (LM) [16,17], which can lead to misidentification. The species *K. papilionacea* and *K. brevis* are morphologically similar and are often present in the same blooms. In an early report, *K. papilionacea* was misidentified as a butterfly shaped *K. brevis* [18]. Subsequently, Haywood et al. described *K. papilionacea* as a separate species and suggested that *K. brevis/K.brevis*-like blooms in Japan may have been misidentified, with *K. papilionacea* being the likely causative species [10]. Molecular sequences of *K. papilionacea* are quite divergent from *K. brevis*, and DNA sequences can, therefore, be useful for distinguishing these species [10]. In addition, the cell shapes of unarmored *Karenia* species have been reported to change rapidly under stressed growth conditions [3,19]. Therefore, the morphological similarity and plasticity of unarmored *Karenia* species makes it more difficult to distinguish and identify them from field samples. Thus, molecular genetic approaches may be useful for the detection and identification of *Karenia* species. 

In Korea, the first major bloom of *Karenia* species that was recorded occurred in Jinhae Bay in August 1981, and the causative organism was identified as *K. mikimotoi* [20,21]. That highly dense bloom (up to 145,000 cells mL^−1^) resulted in oxygen deficiency in the water column, leading to mass mortality of benthic shellfish and mussels. This was the first official report on damage to fisheries caused by the so-called “red tides” in Korea. A second major event that caused damage to fisheries occurred during a bloom involving unidentified *Karenia* species (recorded as *Gyrodinium* sp.) in Tongyeong in August 1992 [22]. The frequency of major *Karenia* blooms, which did not reoccur until 2016, has decreased ever since. *K. mikimotoi* blooms reocurred in southwest Korea and resulted in the mass mortality of cultured abalone in August in 2016 [23]. To date, *K. mikimotoi* has been recognized as the most harmful bloom-forming *Karenia* species in Korean coastal waters. *Karenia papilionacea* was first recorded in Yongho Bay on the southeast coast of Korea in July 2018 [24]. Recently, the neurotoxic brevetoxin (i.e., the ladder frame polyester brevetoxin-2, PbTx-2) was detected in *K. papilionacea*, which is also present in the neurotoxic *K. brevis* [15]. Therefore, it is essential to explore the distribution and dynamics of *K. papilionacea* and to establish a monitoring system for the toxigenic species along the Korean coast. 

The aim of this study was to investigate the distribution and dynamics of *K. papilionacea* and *K. mikimotoi* in coastal waters of Korea, and to determine whether these two species co-occur in time and space. For rapid, sensitive, and accurate detection of these species, we developed a quantitative real-time PCR (qPCR) assay targeting the ITS2 region of the rRNA gene and employed standard curves that were constructed using known concentrations of cultured cells. Subsequently, we employed the developed qPCR assay to examine the distribution and dynamics of the two target species in Korean coastal waters. We also investigated the specific growth rates of *K. papilionacea* and *K. mikimotoi* in laboratory cultures of local strains to evaluate optimal temperature and salinity conditions for bloom formation. These conditions for optimal growth rates were then compared with the distribution and dynamics of the two species in the field. 

## 2. Results

### 2.1. Morphological and Molecular Traits

**Figure 1:**
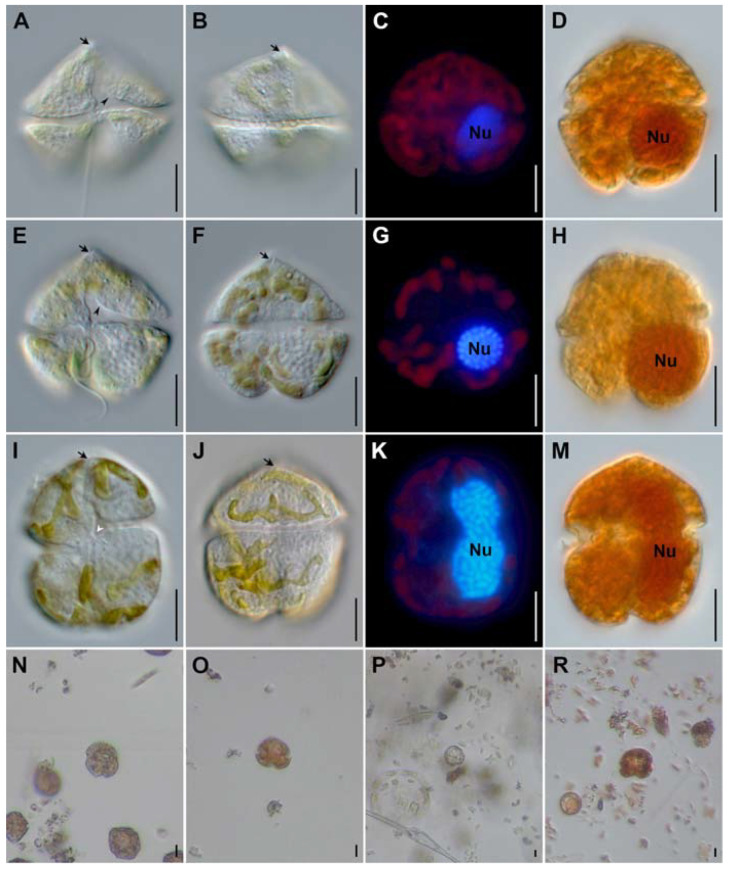
Light micrographs of *Karenia* from laboratory cultures. *Karenia papilionacea* strain LOMME01 (**A**–**D**), *K. papilionacea* strain LOMME02 (**E**–**H**), and *K. mikimotoi* strain LOHABE01 (**I**–**M**). Field specimens of *Karenia* spp. from Lugol-fixed field samples (**N**–**R**). Micrographs of live specimens from cultures observed with differential interference contrast (**A**,**B**,**E**,**F**,**I**,**J**) were made with differential interference contrast; DAPI-stained specimens (**C**,**G**,**K**) with an epifluorescence and Lugol-fixed specimens (**D**,**H**,**M**) with bright field microscopy. Lugol-fixed specimens are from samples collected in Yeosu (**N**), Hakri (**O**), Magumpo (**P**), and Mijo (**R**). Note highlighted cellular features: apical groove in (**A**,**B**,**E**,**F**,**I**,**J**) (arrow); sulcal intrusion open to the epicone in (**E**) (arrowhead); cingulum displacement in (**I**) (white arrowhead); and nucleus (Nu). Scale bars represent 10 µm.

Korean strains of *K. papilionacea* and *K. mikimotoi* from the southern coast of Korea have similar cell size ranges, although *K. papilionacea* cells are slightly wider than *K. mikimotoi* (Table 1). Detailed observations with a light microscope at a high magnification revealed that the two *K. papilionacea* strains have a slightly pointed carina with a short straight apical groove (Figure 1). The Korean *K. papilionacea* strains have a slightly excavated hypotheca, which is less prominent than the excavated, bilobed hypotheca of the typical ‘butterfly’-shaped cells of the originally described New Zealand strain [7]. The sulcal intrusion of *K. papilionacea* is open to the epitheca, whereas *K. mikimotoi* has a closed sulcus (Figure 1). The prominent features of *K. papilionacea* including a pointed carina, apical and sulcal grooves, and sulcal intrusion, were not obvious in Lugol’s iodine-fixed specimens (Figure 1). The nucleus of *K. papilionacea* is spherical to slightly oval and located on the left hypothecal lobe, whereas the nucleus of *K. mikimotoi* is elliptical to elongated (Figure 1).

**Figure 2:**
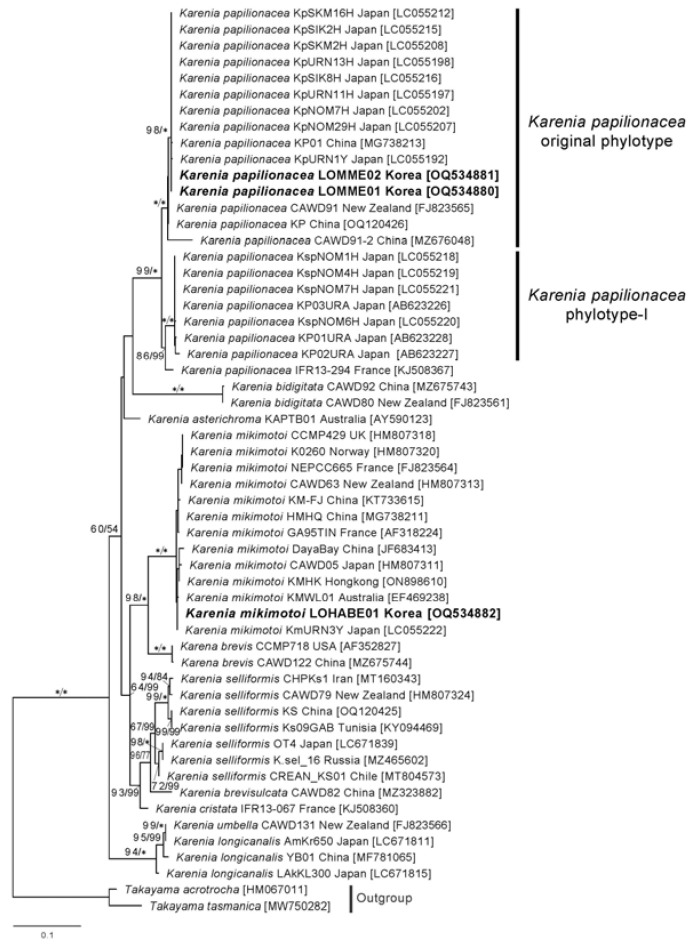
Maximum likelihood tree inferred from ITS sequences of *Karenia* species. Numbers above the nodes represent ML bootstrap supports (left, LBS) and Bayesian posterior probabilities (right, BPP) higher than 60% and 0.7, respectively. Robust statistical supports (100 of LBS or 1.0 of BPP) are indicated by an asterisk (*).

The internal transcribed spacer (ITS) sequences (ITS1, 5.8S rDNA, and ITS2 regions) of the two Korean strains of *K. papilionacea* were identical, and a BLAST search showed that these strains matched the sequences of the Japanese isolates (e.g., KpSKM, KpSIK, KpURN, and KpNOM) with 100% identity. In the maximum likelihood (ML) phylogeny inferred from the ITS sequences, the two Korean strains of *K. papilionacea* nested within the clade of the original *K. papilionacae* phylotype and clustered together with the strains and isolates obtained from Japan, China, and New Zealand, with a strong statistical support of 98/1.0 (Figure 2).

### 2.2. Quantitative PCR Assay Development

#### 2.2.1. Specificity of Primer Pairs for the Two *Karenia* Species

**Figure 3:**
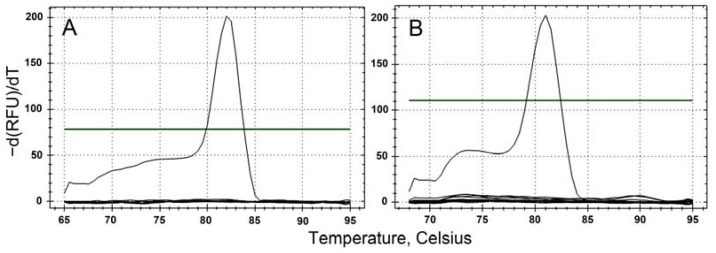
Specificity and accuracy of qPCR assays for the target species, *K. papilionacea* (**A**) and *K. mikimotoi* (**B**).

The specificity of the designed primer pairs targeting the two toxic dinoflagellates, *K. papilionacea* (KpSF-KpSR) and *K. mikimotoi* (KmF-KmR), was determined by performing qPCR assays on target and non-target algal species. Each primer pair amplified only the target species (Table 2). The melting temperature was approximately 81 °C for *K. papilionacea* and 80.5 °C for *K. mikimotoi* (Figure 3). In addition, as a result of sequencing all positive qPCR products, the sequences were found to be 100% identical to the target gene sequences of the two dinoflagellate species.

#### 2.2.2. Accuracy of the Quantitative PCR Assay

**Figure 4:**
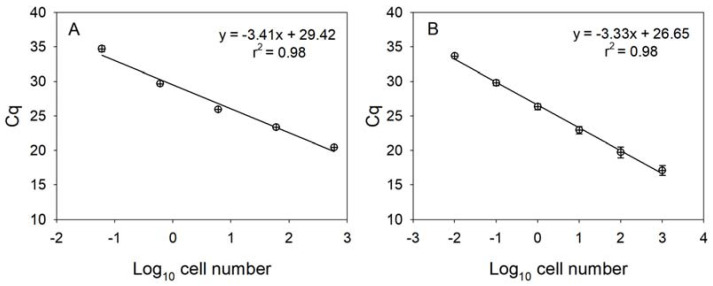
Linear standard curves derived from 10-fold serially diluted DNA purified using known numbers of *K. papilionacea* (**A**) and *K. mikimotoi* (**B**). The lower-case X and *y*-axis represent a log_10_ of cell number and mean (±SE) Cq value, respectively.

Each standard curve for the two *Karenia* species was constructed with ten-fold serial dilutions of the genomic DNAs from *K. papilionacea* (0.06–600 cells) and *K. mikimotoi* (0.1–1000 cells). Strong linear relationships (r^2^ ≥ 0.98) between the Cq values (the mean value in triplicate) and the log of cell numbers for the two *Karenia* species were observed in each standard curve (Figure 4). The reaction efficiencies (*E*) were 96.5% (KpSF/KpSR) and 100.0% (KmF/KmR), respectively, as calculated by the formula *E* = 10^(−1/*S*)^ − 1, where *S* is the slope of the standard curve.

### 2.3. Distributions of K. papilionacea and K. mikimotoi in Korean Coastal Waters

**Figure 5:**
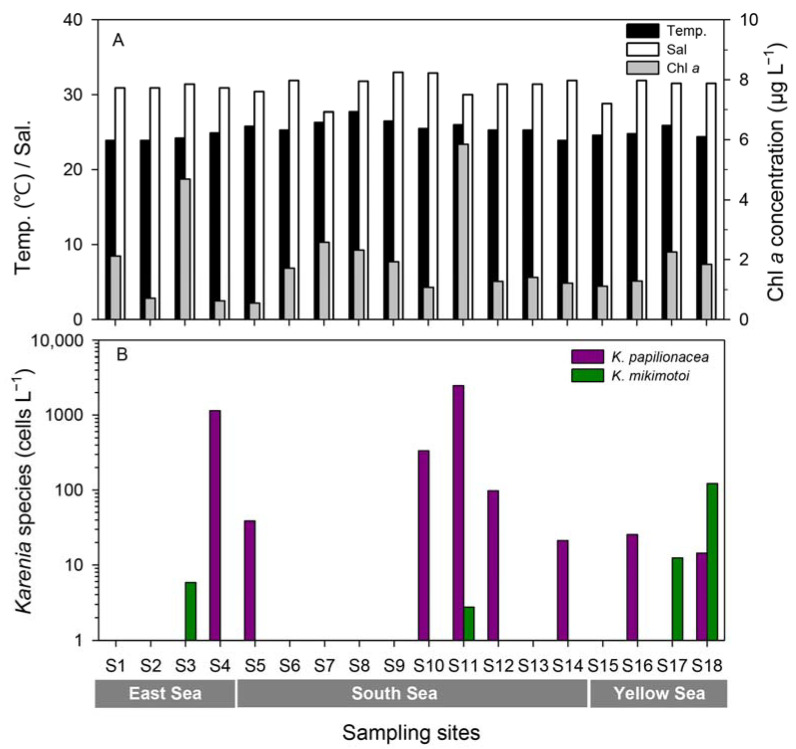
Temperature, salinity, and Chl-*a* concentrations (**A**) and abundances of *K. papilinocea* and *K. mikimotoi* (**B**) at each site along the Korean coast in September 2017.

**Figure 6:**
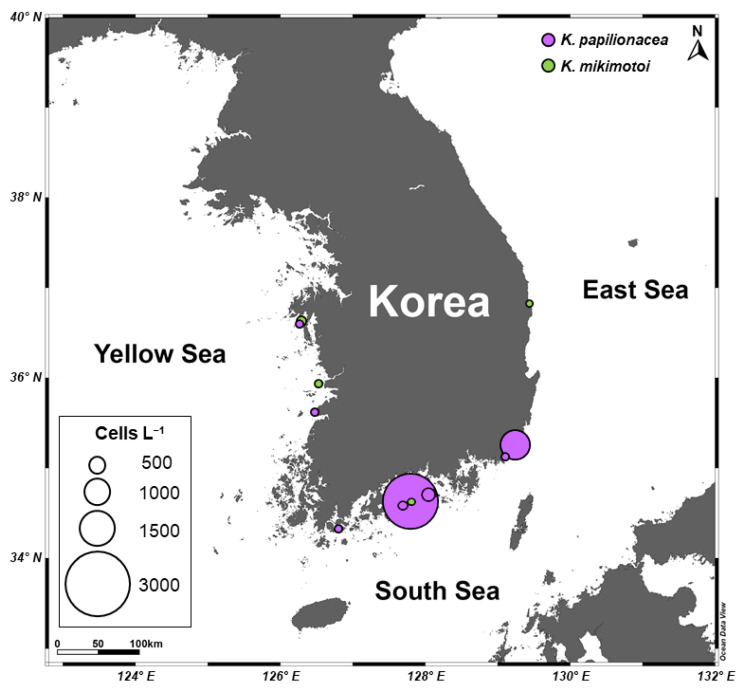
A map showing distributions and abundances of *Karenia mikimotoi* and *K. papilionacea* along the Korean coast in September 2017.

Quantitative estimates of abundance by qPCR of the two species of *Karenia* showed that *K. papilionacea* was distributed in the East Sea (S4), South Sea (S5, S10, S11, S12, and S14), and Yellow Sea (S16 and S18), whereas *K. mikimotoi* was detected in the East Sea (S3), South Sea (S11), and Yellow Sea (S17 and S18) (Figure 5B, Table S1). The abundance of *K. papilionacea* (15–2553 cells L^−1^) was generally higher than that of *K. mikimotoi* (3–122 cells L^−1^) in Korean coastal waters. The abundance of *K. papilionacea* was relatively higher at the sites in the South Sea than in other areas, while *K. mikimotoi* appeared in higher abundances at the sites in the Yellow Sea than in other areas (Figure 6). The two *Karenia* species co-occurred at two sampling sites (S11 and S18), but their abundances were different: at site S11, the abundance of *K. papilionacea* was approximately a thousand-times higher than that of *K. mikimotoi*, whereas *K. mikimotoi* was more abundant at site S18. 

### 2.4. Dynamics of K. papilionacea and K. mikimotoi Populations at a Fixed Station

**Figure 7:**
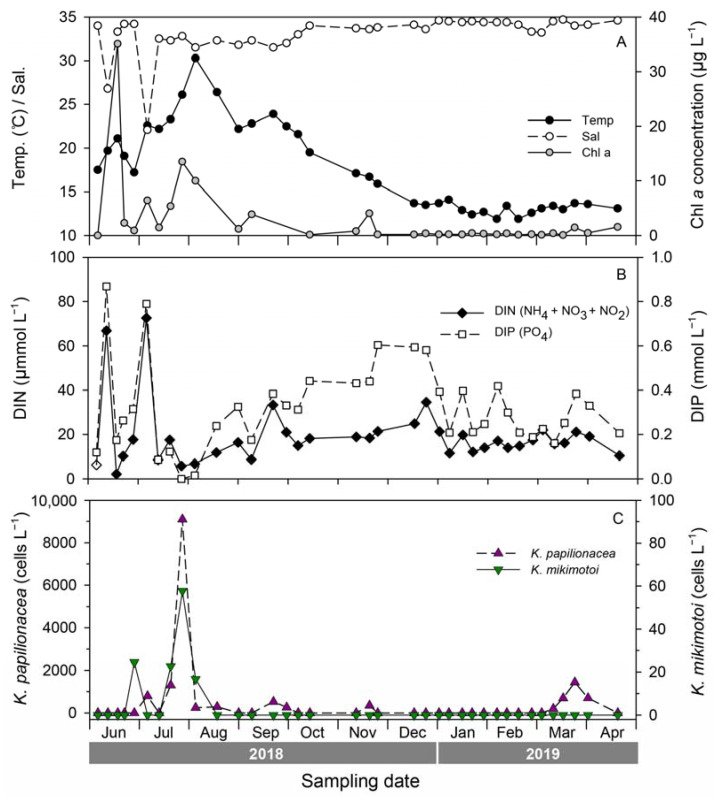
Seasonal variability of environmental factors (**A**), inorganic nutrients (**B**), and abundances of *Karenia papilioncea* and *K. mikimotoi* estimated by a qPCR assay (**C**) at site S5 (Yongho) during weekly monitoring from 2018 to 2019.

To investigate seasonal fluctuations in the two *Karenia* species, weekly monitoring was conducted at S5 from June 2018 to April 2019 (Figure 7). During this period, the water temperature ranged from 11.9 to 30.3 °C, with the highest temperature occurring on 3 August 2018, and the lowest on 1 February 2018. The salinity ranged from 22.1 to 34.7 with sharp drops on June 11 and July 5, 2018, due to heavy rainfall. The chlorophyll-*a* concentration ranged from 0.08 to 35.06 μg L^−1^, with the highest value recorded during an *Akashiwo sanguinea* bloom in June 2018. The abundance of *K. papilionacea* peaked five times during the study; the highest peak (9289 cells L^−1^) was recorded on 26 July 2018, with a water temperature of 26.1 °C and a salinity of 32.8. In the case of *K. mikimotoi*, there were two peaks in summer (June and July), and the highest value was 921 cells L^−1^ on 26 July 2018. Interestingly, both *Karenia* species showed their highest abundance on the same date (26 July 2018). During the study period, the two *Karenia* species appeared under similar environmental conditions; occurrence temperature ranges were 16.7–30.3 °C for *K. papilionacea* and 17.2–30.3 °C for *K. mikimotoi*, and the corresponding salinity ranges were 22.1–33.6 and 31.5–34.2, respectively (Figure 7). 

### 2.5. Growth Responses of K. papilionacea and K. mikimotoi Cultures to Temperature and Salinity

**Figure 8:**
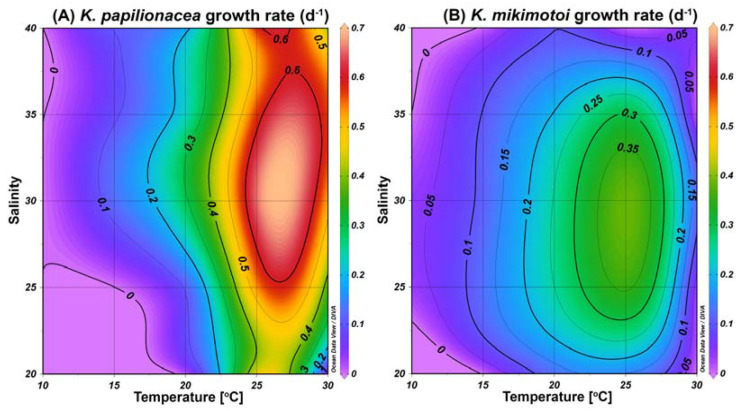
Specific growth rates (d^−1^) of *K. papilionacea* Lomme01 (**A**) and *K. mikimotoi* (**B**) as a function of water temperature (°C) and salinity.

**Figure 9:**
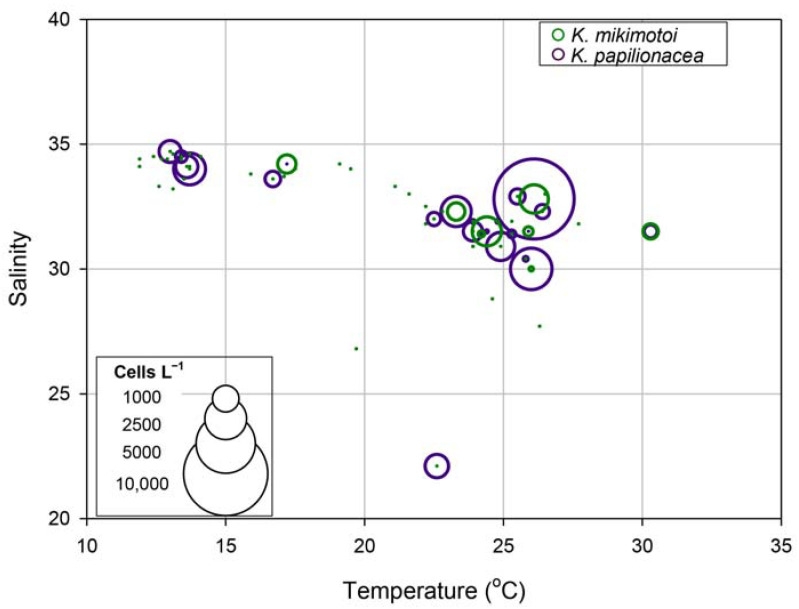
Relationships among temperature, salinity, and cell abundances (cells L^−1^) of *Karenia papilionacea* and *K. mikimotoi* in Korean coastal waters.

To understand the similar eco-physiological traits of the two species in the field, we investigated their growth responses under different temperature and salinity conditions in cultures. The highest specific growth rate of *K. papilionacea* was observed at a temperature of 25 °C and a salinity of 30 (Figure 8). This dinoflagellate was able to grow well at higher temperatures (25 and 30 °C) regardless of the salinity level. The growth rate of *K. mikimotoi* was generally lower than that of *K. papilionacea* (Figure 8). Notably, in addition to the field observations, both *Karenia* species showed similar physiological responses to temperature and salinity (Figure 9). 

## 3. Discussion

Due to the similar morphologies of *Karenia species*, it is difficult to distinguish them precisely in field samples under an LM [10], and the development of an alternative method is therefore necessary. qPCR assays can accurately enumerate algal cell numbers [25,26,27], and have been widely used in field studies, including algal population dynamics studies [28,29]. As the first step in developing a suitable qPCR assay, we used specific primer pairs targeting the ITS2 region of the rRNA gene for each *Karenia* species. The ITS region, a component of the rRNA gene, is commonly used for species-level identification because of its high degree of variation among species [30,31] and, thus, is useful for designing specific primer pairs [25,32]. To evaluate the specificity of the developed primer pairs, a cross-reactivity test was performed using algal cultures with similar sequences to the two *Karenia* species. The results of this test showed that specific primer pairs only amplified the target species. Based on these findings, each specific primer pair was appropriate for use in the qPCR assay to detect and quantify the target species.

Based on our findings, all standard curves for the target *Karenia* species were highly significant and the reaction efficiencies were also adequate, indicating that the designed primer pair ideally amplified the target DNA. Additionally, melting curve analysis was performed to determine the presence of unexpected amplicons or primer dimers. All melting curves displayed a single informative and narrow peak, suggesting successful amplification of the target region without nonspecific amplification. Together with these findings, the qPCR assay based on the designed primer pairs can accurately detect and quantify the two *Karenia* species, and we successfully applied this assay to the field samples. However, the presence of PCR inhibitors [25], such as mucopolysaccharides, phenolic compounds, humic acids, and heavy metals [9,33,34,35,36], in the field samples can largely reduce qPCR efficiency, leading to a decrease in the accuracy of qPCR measurements. Here, we used the dilution method described by Park et al. [25] to reduce the effect of PCR inhibitors based on an optimal dilution factor. We evaluated the removal of PCR inhibitors by assessing the PCR efficiency of the field samples after dilution. As a results, there was no effect of PCR inhibitors on the amplification reaction when the field samples were diluted by a factor of 20 with a reaction efficiency of 98.7 ± 3.5% (average ± standard deviation, *n* = 20). Therefore, the abundances of the two *Karenia* species in the field samples were measured via qPCR assay after a twenty-fold dilution.

Since *K. papilionacea* is morphologically similar to other *Karenia* species, such as *K. brevis*, it can be difficult to precisely identify this dinoflagellate using a microscopic observation [16,17]. Thus, despite its wide distribution [31], the distribution of *K. papilionacea* in Korean coastal waters remained uncertain until our study. In our study, using the qPCR assay, we have identified the presence of *K. papilionacea* in Korean coastal waters for the first time. This molecular technique is useful for the precise detection and quantification of morphologically cryptic species. Additionally, microscopic observation techniques have limitations in detecting and distinguishing similar-looking algal cells especially at low densities, whereas the qPCR assay is highly sensitive and accurate, enabling precise detection and quantification even at low algal densities [25,37]. Based on our findings, *K. papilionacea* is widely distributed in Korean coastal waters, but were mostly at low densities (<100 cells L^−1^). Hence, the qPCR assay could be a useful tool for studying the dynamics of *K. papilionacea* in Korean coastal waters. 

During our study period, the two *Karenia* species exhibited similar dynamics and were occasionally co-distributed. Interestingly, the abundances of the two species were positively correlated with a statistical significance (Pearson r = 0.820, *p* = 0.001). Additionally, in physiological experiments using algal strains, both species displayed similar physiological responses to temperature and salinity. This indicates that *K. papilionacea* and *K. mikimotoi* may have similar eco-physiological characteristics, and this may facilitate the formation of a co-bloom by these dinoflagellates, inducing a synergistic adverse effect on marine environments. 

Although the dynamics of the two target *Karenia* species were similar in the field during the study period, the cell densities of *K. mikimotoi* were generally lower than those of *K. papilionacea*. In our laboratory algal cultures, both species displayed similar physiological characteristics, but *K. papilionacea* exhibited a broader range of environmental conditions for its growth than that of *K. mikimotoi*. In addition, the growth rate of *K. mikimotoi* was generally lower than that of *K. papilionacea*. Taken together, the combination of a higher growth rate and a broader range of growth conditions likely explain the higher cell numbers of *K. papilionacea* when the two species coexist in the field.

Based on a previous study [38], it has been shown that qPCR measurements of dinoflagellates may not be accurate due to the variability in the rRNA gene copy number depending on the species or strain. To determine the rRNA gene copy variability in the target *Karenia* species, we used two *K. papilionacea* strains (Kp-Lomme01 and Kp-Lomme02) and compared their rRNA gene copy numbers (Table S2). The cell numbers of Kp-Lomme01 were calculated using direct counting, and a standard curve was created by plotting with Kp-Lomme02. The cell numbers of Kp-Lomme01 were similar between both assays (within 30% difference). This shows that the qPCR assay developed in this study is capable of detecting and quantifying *K. papilionacea* in the field samples.

It is highly useful to develop a qPCR assay that allows us to separately detect and quantify the two *Karenia* species, which are difficult to distinguish under an LM. Based on our findings, this qPCR assay is reliable for quantifying target species in field samples. For example, the two measurements (direct counting vs. qPCR assay) exhibited not only similarity, but also a significant linear correlation (Pearson r = 0.980, *p* = 0.01). Through the application of the qPCR assays to field samples, we reported the distribution and dynamics of *K. papilionacea* in Korean coastal waters for the first time. During a year-long monitoring period, *K. papilionace* exhibited similar dynamics to *K. mikimotoi*, and these two species had similar physiological characteristics in both field and laboratory observations. Based on these findings, *K. papilionace* and *K. mikimotoi* can proliferate under similar environmental conditions, leading to the formation of co-blooms. As it remains unclear whether co-blooms of the two different toxin-producing *Karenia* species might have a synergistic adverse effect on marine environments, further studies are required in Korean coastal waters.

## 4. Materials and Methods

### 4.1. Cell Cultures

Two strains of *K. papilionacea* (strains Kp-Lomme01 and Kp-Lomme02) were established as clonal cultures by isolation using a glass capillary pipet from a net sample taken from the surface seawater of Yongho Bay in Busan, Korea (35°08′00″ N, 129°06′55″ E) on 24 July 2018, and 24 June 2021, respectively (Table 2). *Karena mikimotoi* (strain LOHABE01) was collected from Hadong, Korea, on 25 August 2015 (Table 2). All stock cultures were grown in salinity 30 f/2-Si medium [39] at 20 °C with a 14:10 h of light:dark cycle of cool-white fluorescent light at 150 μmol photons m^−2^ s^−1^. 

### 4.2. Light Microscopy

Live or Lugol-fixed specimens were observed using an Axio Imager A2 (Carl Zeiss Inc., Oberkochen, Germany) equipped with epifluorescence and differential interference contrast optics. Light micrographs were taken at ×400 and ×1000 magnifications using a photomicrographic system (AxioCam HRc, Carl Zeiss Inc.) coupled to the microscope. Cell size was measured from the micrographs of at least 30 live specimens using AxioVision SE64 Rel.4.9.1 (Carl Zeiss Inc.). To determine the shape and location of nuclei, *Karenia* cells were fixed with glutaraldehyde (final concentration 1%) and then stained with 4′-6-diamidino-2-phenylindole (DAPI: 0.1 μg mL^−1^ final concentration) under an epifluorescence microscope with ultraviolet light (excitation of 360 nm and emission of 460 nm).

### 4.3. Sequences of ITS1, 5.8S rDNA, and the ITS2 Region

Five to ten milliliters of dense *K. papilionacea* and *K. mikimotoi* cultures (approximately 5000–10,000 cells mL^−1^) in the exponential growth phase were pelleted by centrifugation at 13,000 rpm for 5 min. DNA was extracted from the cell pellets using 10% Chelex buffer [40]. PCR amplification was conducted with a 20 μL reaction mixture containing 2 μL template DNA, 0.2 μM primers (ITSA and ITSB) [41], and an AccuPower^®^ PCR premix kit (Bioneer Inc., Daejeon, Korea). The reactions were run using a C1000 TouchTM Thermal Cycler (Bio-Rad, Foster City, CA, USA) under the following conditions: an initial denaturing step at 95 °C for 4 min, followed by 35 cycles (95 °C for 45 s, 55 °C for 45 s, and 72 °C for 2 min), with a final extension at 72 °C for 10 min. PCR products were visualized on EcoDyeTM (SolGent Co., Daejeon, Korea), stained 1% agarose gel, purified using a AccuPrep^®^ Purification kit (Bioneer), and sequenced with PCR primers using a Big-Dye Terminator v3.1 Cycle Sequencing Kit (Applied Biosystems, Foster City, CA, USA) and an ABI model 3730XL DNA analyzer (Applied Biosystems). ContigExpress (Vector NTI ver. 10.1; Invitrogen, Grand Island, NY, USA) was used to edit low quality regions and assemble sequence reads. The assembled sequences were confirmed by a BLASTN search of the NCBI database and deposited in GenBank (accession nos. OQ534880–OQ534882). 

### 4.4. qPCR Assay for the Two Karenia Species

#### 4.4.1. Specific Primer Pairs for the Two *Karenia* Species

Specific primer pairs for *K. papilionacea* for the qPCR assay were designed based on the alignment of the ITS1/5.8S rDNA/ITS2 sequences, including the original type and phylotype I of *K. papilionacea* [31] and other closely related dinoflagellates (30 genera) retrieved from GenBank. Based on the alignment, the specific primer pair for *K. papilionacea* was manually designed to target the ITS2 region (amplicon size: 166 base pairs [bp]) as follows: KpSF (forward primer, 5′-TTG TCT ACA ACT TTG GGT GG-3′) and KpSR (reverse primer, 5′-GCT GAA AGT TGT ATG AAG CAA T-3′). For *K. mikimotoi*, we used a specific primer pair (KmF and KmR) previously reported by Vandersea et al. [7]. The specificity of this primer pair was evaluated in silico using BLAST. Cross-reactivity tests were performed using a qPCR assay with purified DNA extracts of other phytoplankton species that are commonly found in *Karenia* species in Korean coastal waters (Table 2). 

A qPCR assay was performed with triplicate 15 µL reactions containing 2 µL of genomic DNA, 7.5 µL of 1X SsoFastTMEvaGreen Supermix (Bio-Rad, CA, USA), 0.2 µM of each forward and reverse primer, and DNase free water (Bioneer, Korea). The qPCR reaction was run on the CFX Connect Real-Time PCR Detection System (Bio-Rad, CA, USA) with the following thermal cycling conditions: 98 °C for 3 min, followed by 39 cycles of 98 °C for 10 s and 63 °C for 10 s. To assess whether or not this primer pair is capable of amplifying only the target region, a melting curve analysis was conducted from 65 °C to 95 °C with reads every 0.5 °C for 5 s. Additionally, the qPCR products were checked by 2% agarose gel electrophoresis and then sequenced and matched to the sequences of the target species.

#### 4.4.2. qPCR Assay for Standard Curves Construction

To construct the standard curves, *Karenia* cells in the exponential growth phase were harvested between 9 and 11 a.m. to minimize variability in rRNA gene copies due to the diurnal cell cycle [25]. Cell numbers were measured in triplicate by direct counting using a Sedgwick–Rafter chamber under an Axio Imager A2 microscope (Carl Zeiss, Oberkochen, Germany). Triplicate samples consisting of 5 × 10^4^ cells for *K. mikimotoi* and 3 × 10^4^ cells for *K. papilionacea* were added into a 2-mL microtube (Axygen, CA, USA) and harvested by centrifugation at 13,000 rpm for 5 min, and the supernatant was discarded. Next, 800 μL of DNA EX buffer (100 mM Tris-HCl, 100 mM Na_2_-EDTA, 100 mM sodium phosphate, 1.5 M NaCl, and 1% CTAB) was added to the tube and stored at −80 °C until DNA extraction. The pellet was thawed in a 65 °C water bath, and then 8 μL of proteinase K (10 mg mL^−1^) was added to each sample and incubated at 37 °C for 30 min. Following incubation, 80 μL of 20% sodium dodecyl sulfate (SDS) was added to each sample and incubated at 65 °C for 2 h with gentle shaking. Subsequently, 888 μL of chloroform-isoamyl alcohol (24:1) was added to each tube and centrifuged at 10,000× *g* and 20 °C for 5 min. The supernatant of the mixture was transferred to a 2-mL tube, and 88.8 μL of 3 M sodium acetate and 532.8 μL of isopropanol (≥99%) were added. After centrifugation at 14,000× *g* for 20 min, the supernatant was decanted, 1 mL of cold 70% ethanol was added, and DNA was pelleted by centrifugation at 14,000× *g* for 15 min. The pellets were dried at room temperature and dissolved in 100 μL of TE buffer. The purity and quantity of DNA were checked using a NanoDrop ND-1000 system (Thermo Fisher Scientific, DE, USA). The DNA extracts were serially ten-fold diluted and used to generate the standard curves. Dilution ranged from 0.03 to 300 cells μL^−1^ for *K. papilionacea* and from 0.05 to 500 cells μL^−1^ for *K. mikimotoi*. qPCR for the construction of the two *Karenia* species was conducted as described in Section 4.4.1.

#### 4.4.3. Comparison of rRNA Gene Copies for *Karenia* Species

To examine rRNA gene copy variability among *K. papilionacea* strains, we modified the protocol described by Park et al. [25]. The strain Lomme02 was counted in a Sedgewick–Rafter (SR) chamber under an LM. Lomme02 aliquots were pelleted, and genomic DNA was extracted as described in Section 4.4.2. A qPCR assay was performed with duplicate 15 µL reactions under the same thermal cycling conditions as previously described. The rRNA gene copy variability among the strains was inferred by comparing the cell number of strain Lomme02 measured by direct cell counting with that estimated from the qPCR assay based on the standard curve generated with strain Lomme01.

### 4.5. Distribution and Bloom Dynamics

#### 4.5.1. Sampling

**Figure 10:**
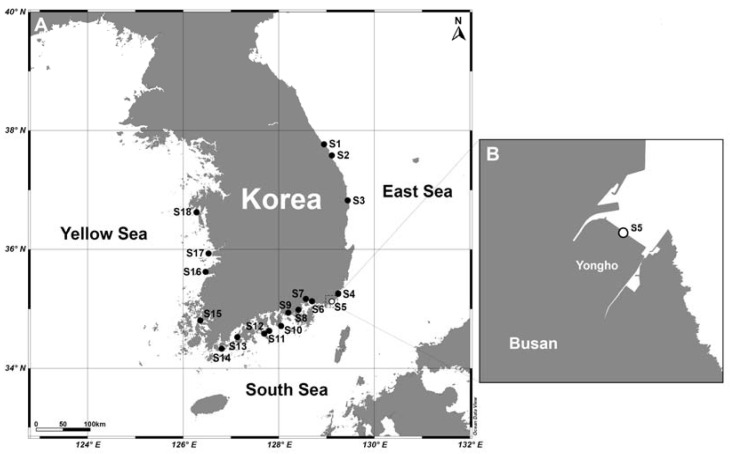
Map showing sampling sites during a nationwide survey along Korean coastal waters in September 2017 (**A**). Weekly monitoring was conducted at site 5 (S5) from 2018 to 2019 (**B**).

Samples were collected from surface seawater at 18 sites in Korean coastal waters in September 2017 (Table S1, Figure 10). To assess the seasonal dynamics of the two *Karenia* species, weekly monitoring was also performed from June 2018 to April 2019 at a fixed site (S5, Yongho Bay, Busan) (Table S1, Figure 10). Surface seawater temperature and salinity were measured in situ using a YSI 300 instrument (YSI Inc., Yellow Springs, OH, USA). To determine Chl-*a* concentrations, 100 mL of seawater were filtered onto 47 mm GF/F filters. Each filter was soaked in 90% acetone for 24 h at 4 °C in the dark. The extracts were measured using a 10-AU fluorometer (Turner Design). For the analysis of inorganic nutrients (NO_3_, NO_2_, NH_4_, and PO_4_), aliquots of the GF/F filtrate were stored at −20 °C until analysis. Inorganic nutrient concentrations were measured using an AutoAnalyzer (QuAAtro, Seal Analytical, Inc., Norderstedt, Germany). 

For qPCR analyses, 250 mL of surface seawater was filtered onto a 3.0µm pore-sized polycarbonate filter (Isopore^TM^ Membrane, Merck, Lebanon, NJ, USA). The filter was placed in a 2-mL microtube containing 800 μL of DNA EX buffer (100 mM Tris-HCl, 100 mM Na2-EDTA, 100 mM sodium phosphate, 1.5 M NaCl, 1% CTAB) stored at −80 °C until DNA extraction. 

#### 4.5.2. qPCR Assay for Environmental Samples

The DNA from the fields samples was extracted using the same protocol as described in Section 4.4.2. As various PCR inhibitors are present in the field samples, we determined an optimal dilution factor (20-fold) by examining the PCR efficiency of the field samples after dilution. The abundance of the two *Karenia* species in the diluted field samples was measured using qPCR assays, and the distribution of these dinoflagellates in Korean coastal waters was identified. To determine the abundance of *K. papilionacea* and *K. mikimotoi* in the field samples, a qPCR assay was conducted using specific primers under the same thermal cycle conditions as described in Section 4.4.1. The DNA from each sample was amplified three times to ensure the accuracy of the results. Negative control reactions were performed using DNase-free water. The products from all qPCR reactions were run on a 2% agarose gel via electrophoresis to determine whether the PCR products were of the expected length. The products were then purified with ExoSAP-IT (Affymetrix) and Sanger sequenced using the same primers as used for qPCR. The resulting sequences were verified using a BLASTN search. 

### 4.6. Growth Response of Karenia Species to Temperature and Salinity

Stock cultures of *K. papilionacea* (Kp-Lomme01) and *K. mikimotoi* were grown at 20 °C with a salinity of 30 with a light:dark cycle of 14:10 h. The growth experiments were compared using a crossed factorial design with 25 combinations of five temperatures (10, 15, 20, 25, and 30 °C) and five salinities (20, 25, 30, 35, and 40) using an incubator (SW-90B2, Gaon Sci, Korea). The five salinity gradients were adjusted by adding modified media of salinities of 0 and 47; the salinity of 0 was made with sterile deionized water, and the salinity of 47 was made by evaporating sterile 20 μm-filtered seawater. A total of 25 flasks contained 50 mL of the stock cultures and five flasks were placed into each of the five growth chambers. Cultures were acclimated to the desired experimental conditions by stepwise changes in salinity and temperature of 2.5 and 1 °C per day, respectively. All cultures—adapted to each condition—were adjusted to a final concentration of 100 cells mL^−1^, and then distributed into 10-mL glass culture tubes in triplicate (Kimble Chase, Rockwood, TX, USA). Each tube measured the in vivo chlorophyll fluorescence using a fluorometer (10-AU, Turner Designs, USA) every day with stabilization for 10 min in the dark before the measurement. The growth responses of the target *Karenia* species under each condition were estimated from the relationships between cell abundance and in vivo chlorophyll fluorescence (Figure S1). Specific growth rates during the exponential growth phase were calculated using the method of Guillard [42]; μ (d^−1^) = ln (N_2_/N_1_)/Δt, where N_2_ and N_1_ are the cell numbers at the end (t_2_) and beginning (t_1_) of a period of time, and Δt is the t_2_−t_1_. 

### 4.7. Statistical Analyses

All statistical analyses were performed using SPSS version 21 software (SPSS, Inc., Chigaco, IL, USA). Pearson correlation coefficient was used to examine the statistical correlation between the following variables: (i) direct counting and qPCR measurements for both *Karenia* species in the field, and (ii) abundances of *K. papilionacea* and *K. mikimotoi* in the field during the study period.

## Figures and Tables

**Table 1:** Morphometry of *K. mikimotoi* (LOHABE01) and the two strains of *K. papilionacea* (Kp-Lomme01/Kp-Lomme02) isolated and cultivated during this study.

Species	Strain No.	Origin		Cell Size	
		Location	Isolation Date	Length (Range) (Mean ± SE)	Width (Range) (Mean ± SE)
*K. mikimotoi*	LOHABE01	Hadong, Namhae (South Sea)	11 Aug. 2015	25.2–35.2 (30.2 ± 0.5)	20.2–31.4 (25.9 ± 0.5)
*K. papilionacea*	Kp-Lomme01	Yongho, Busan (South Sea)	24 Jul. 2018	22.4–38.5 (29.1 ± 0.8)	22.9–44.9 (30.9 ± 1.0)
	Kp-Lomme02	Yongho, Busan (South Sea)	24 Jun. 2021	20.7–33.1 (27.5 ± 0.6)	20.0–37.3 (28.4 ± 0.9)

**Table 2:** Specificity of the qPCR primer pairs for *Karenia* species and closely related dinoflagellate species.

Species	Strain No.	KpSF/KpSR	KmF/KmR
*Akashiwo sanguinea*	As-Lomme09	-	-
*Alexandrium affine*	Aa-Lomme04	-	-
*Al. pacificum*	Ap-Lomme07	-	-
*Biecheleriopsis adriatica*	Ba-Lomme01	-	-
*Gonyaulax whaseongensis*	Gw-Lomme02	-	-
*Gymnodinium aureolum*	Ga-Lomme01	-	-
*Gy. impudicum*	Gi-Lomme01	-	-
*Heterocapsa minima*	Hm-Lomme01	-	-
*Karenia mikimotoi*	Km-Lohabe01	-	Pos
*K. papilionacea*	Kp-Lomme01	Pos	-
*K. papilionacea*	Kp-Lomme02	Pos	-
*Karlodinium digitatum*	Kd-Lomme01	-	-
*Karl. veneficum*	Kv-Lomme03	-	-
*Karl. zhouanum*	Kz-Lomme01	-	-
*Levanderina fissa*	Lf-Lomme01	-	-
*Prorocentrum micans*	Pm-Lomme01	-	-
*Takayama acrotrocha*	Ta-Lomme01	-	-
*T. tasmanica*	Tt-Lomme01	-	-
*Scrippsiella masanensis*	Sm-Lomme01	-	-

## Data Availability

Not applicable.

## Supplementary Materials

The following supporting information can be downloaded at https://www.mdpi.com/article/10.3390/toxins15070469/s1, Table S1: List of sampling locations, sampling dates, temperature, salinity, cell abundances (cells L^−1^) of *K. papilionacea* (Kp) and *K. mikimotoi* (Km) estimated by qPCR assay at each site in Korean coastal waters in September 2017. Table S2: Calculated cell number (per mL) by direct counting and quantitative real-time PCR assay for comparison of cell numbers between strains. qPCR values represent mean ± standard error (SE). Figure S1: Relationship between cell abundance and in vivo chlorophyll fluorescence in *K. papilionacea* (A) and *K. mikimotoi* (B).

## References

[1] Tester P.A., Steidinger K.A. (1997). *Gymnodinium breve* red Tide Blooms: Initiation, Transport, and Consequences of Surface Circulation. Limnol. Oceanogr..

[2] Landsberg J.H., Steidinger K.A., Reguera B., Blanco J., Fernandez M.L., Wyatt T. (1998). A Historical Review of *Gymnodinium breve* Red Tides Implicated in Mass Mortalities of the Manatee (*Trichechus manatus latirostris*) in Florida, USA. Harmful Algae.

[3] Steidinger K.A., Stockwell D.A., Truby E.W., Wardle W.J., Dortch Q., Van Dolah F.M. (1998). Phytoplankton Blooms off Louisiana and Texas, May–June 1994. NOAA Tech. Rep. NMFS.

[4] Tester P.A., Turner J.T., Shea D. (2000). Vectorial Transport of Toxins from the Dinoflagellate *Gymnodinium breve* through Copepods to Fish. J. Plankton Res..

[5] Satake M., Shoji M., Oshima Y., Naoki H., Yasumoto T. (2002). Gymnocin-A, a cytotoxic polyether from the notorious red tide dinoflagellate, *Gymnodinium mikimotoi*. Tetrahedron Lett..

[6] Chang F.H., MacKenzie L., Till D., Hannah D., Rhodes L., Lassus P., Arzul G., Erard E., Gentien P. (1995). The First Toxic Shellfish Outbreaks and the Associated Phytoplankton Blooms in Early 1993 in New Zealand. Harmful Marine Algal Blooms.

[7] Vandersea M., Tester P., Holderied K., Hondolero D., Kibler S., Powell K., Baird S., Doroff A., Dugan D., Meredith A. (2020). An Extraordinary *Karenia mikimotoi* “Beer Tide” in Kachemak Bay Alaska. Harmful Algae.

[8] Yasumoto T., Underal B., Aune T., Hormazabal V., Skulberg O.M., Oshim S. (1990). Screening for Hemolytic and Ichthyotoxic Components of *Chrysochromulina* spp. and *Gyrodinium aureolum* from Norwegian Coastal Waters. Toxic Mar. Phytoplankton.

[9] Parrish C.C., Bodennec G., Gentien P. (1998). Haemolytic Glycoglycerolipids from *Gymnodinium* Species. Phytochemistry.

[10] Haywood A.J., Steidinger K.A., Truby E.W., Bergquist P.R., Bergquist P.L., Adamson J., Mackenzie L. (2004). Comparative Morphology and Molecular Phylogenetic Analysis of Three New Species of the Genus *Karenia* (*Dinophyceae*) from New Zealand: Three New Species of *Karenia*. J. Phycol..

[11] Seki T., Satake M., Mackenzie L., Kaspar H.F., Yasumoto T. (1995). Gymnodimine, a New Marine Toxin of Unprecedented Structure Isolated from New Zealand Oysters and the Dinoflagellate, *Gymnodinium* sp. Tetrahedron Lett..

[12] Holland P.T., Shi F., Satake M., Hamamoto Y., Ito E., Beuzenberg V., McNabb P., Munday R., Briggs L., Truman P. (2012). Novel Toxins Produced by the Dinoflagellate *Karenia brevisulcata*. Harmful Algae.

[13] Heil C.A., Steidinger K.A. (2009). Monitoring, Management, and Mitigation of *Karenia* Blooms in the Eastern Gulf of Mexico. Harmful Algae.

[14] Haywood A., Inoguchi N., Mackenzie L., Garthwaite I., Towers N. (1996). *Gymnodinium breve* “Look-Alikes”: Three *Gymnodinium* Isolates from New Zealand. Harmful Toxic Algal Bloom..

[15] Fowler N., Tomas C., Baden D., Campbell L., Bourdelais A. (2015). Chemical analysis of *Karenia papilionacea*. Toxicon.

[16] Steidinger K., Babcock C., Mahmoudi B., Tomas C., Truby E., Okaichi T., Anderson D.M., Nemoto T. (1989). Conservative Taxonomic Characters in Toxic Dinoflagellate Species Identification. Red Tides: Biology, Environmental Science, and Toxicology.

[17] Taylor F.J.R. (1992). The Taxonomy of Harmful Marine Phytoplankton. G. Bot. Ital..

[18] Steidinger K.A. (1979). Collection, Enumeration and Identification of Free-Living Marine Dinoflagellates [Algae]. Dev. Mar. Biol..

[19] Gomez F. (2006). The Dinoflagellate Genera *Brachidinium*, *Asterodinium*, *Microceratium* and *Karenia* in the Open SE Pacific Ocean. Algae.

[20] Cho C.-H. (1981). On the *Gymnodinium* Red Tide in Jinhae Bay. Hangug Susan Haghoi Ji.

[21] Park J.S., Kim H.G., Lee S.G. (1988). Red Tide Occurrence and Succession of Its Causative Organisms in Jinhae Bay. Bull. Nat. Fish. Res. Dev. Agency.

[22] Kim H.G., Park J.S., Lee S.G., An K.H. (1993). Illustration of Plankton Responsible for the Blooms in Korean Coastal Waters.

[23] Lim W.A., Go W.J., Kim K.Y., Park J.W. (2020). Variation in Harmful Algal Blooms in Korean Coastal Waters Since 1970. J. Korean Soc. Mar. Environ. Saf..

[24] Cho M., Choi H., Nam S.W., Kim S. (2021). Newly Recorded Unarmored Dinoflagellates in the Family Kareniaceae (*Gymnodiniales*, *Dinophyceae*) in Brackish and Coastal Waters of Korea. Korean J. Environ. Biol..

[25] Park B.S., Wang P., Kim J.H., Kim J.-H., Gobler C.J., Han M.-S. (2014). Resolving the Intra-Specific Succession within *Cochlodinium polykrikoides* Populations in Southern Korean Coastal Waters via Use of Quantitative PCR Assays. Harmful Algae.

[26] Kim J.-H., Kim J.H., Wang P., Park B.S., Han M.-S. (2016). An Improved Quantitative Real-Time PCR Assay for the Enumeration of *Heterosigma akashiwo* (Raphidophyceae) Cysts Using a DNA Debris Removal Method and a Cyst-Based Standard Curve. PLoS ONE.

[27] Kim J.H., Kim J.-H., Park B.S., Wang P., Patidar S.K., Han M.-S. (2017). Development of a QPCR Assay for Tracking the Ecological Niches of Genetic Sub-Populations within *Pseudo-nitzschia pungens* (*Bacillariophyceae*). Harmful Algae.

[28] Park B.S., Kim J.H., Kim J.-H., Baek S.H., Han M.-S. (2018). Intraspecific Bloom Succession in the Harmful Dinoflagellate *Cochlodinium polykrikoides* (Dinophyceae) Extended the Blooming Period in Korean Coastal Waters in 2009. Harmful Algae.

[29] Kim J.H., Wang P., Park B.S., Kim J.-H., Patidar S.K., Han M.-S. (2018). Revealing the Distinct Habitat Ranges and Hybrid Zone of Genetic Sub-Populations within *Pseudo-nitzschia pungens* (*Bacillariophyceae*) in the West Pacific Area. Harmful Algae.

[30] Gardes M., Bruns T.D. (1993). ITS Primers with Enhanced Specificity for Basidiomycetes-Application to the Identification of Mycorrhizae and Rusts. Mol. Ecol..

[31] Yamaguchi H., Hirano T., Yoshimatsu T., Tanimoto Y., Matsumoto T., Suzuki S., Hayashi Y., Urabe A., Miyamura K., Sakamoto S. (2016). Occurrence of *Karenia papilionacea* (Dinophyceae) and Its Novel Sister Phylotype in Japanese Coastal Waters. Harmful Algae.

[32] Park B.S., Kim S., Kim J.-H., Ho Kim J., Han M.-S. (2019). Dynamics of Amoebophrya Parasites during Recurrent Blooms of the Ichthyotoxic Dinoflagellate *Cochlodinium polykrikoides* in Korean Coastal Waters. Harmful Algae.

[33] Audemard C., Reece K.S., Burreson E.M. (2004). Real-Time PCR for Detection and Quantification of the Protistan Parasite *Perkinsus marinus* in Environmental Waters. Appl. Environ. Microbiol..

[34] Audemard C., Ragone Calvo L.M., Paynter K.T., Reece K.S., Burreson E.M. (2006). Real-Time PCR Investigation of Parasite Ecology: In Situ Determination of Oyster Parasite *Perkinsus marinus* Transmission Dynamics in Lower Chesapeake Bay. Parasitology.

[35] Vargas-Montero M., Freer E., Jiménez-Montealegre R., Guzmán J.C. (2006). Occurrence and Predominance of the Fish Killer *Cochlodinium polykrikoides* on the Pacific Coast of Costa Rica. Afr. J. Mar. Sci..

[36] Faveri J.D., Smolowitz R.M., Roberts S.B. (2009). Development and Validation of a Real-Time Quantitative PCR Assay for the Detection and Quantification of *Perkinsus marinus* in the Eastern Oyster, Crassostrea Virginica. J. Shellfish Res..

[37] Park B.S., Baek S.H., Ki J.-S., Cattolico R.A., Han M.-S. (2012). Assessment of EvaGreen-Based Quantitative Real-Time PCR Assay for Enumeration of the Microalgae *Heterosigma* and *Chattonella* (*Raphidophyceae*). J. Appl. Phycol..

[38] Galluzzi L., Bertozzini E., Penna A., Perini F., Garcés E., Magnani M. (2010). Analysis of rRNA Gene Content in the Mediterranean Dinoflagellate *Alexandrium catenella* and *Alexandrium taylori*: Implications for the Quantitative Real-Time PCR-Based Monitoring Methods. J. Appl. Phycol..

[39] Guillard R.R.L., Ryther J.H. (1962). Studies of Marine Planktonic Diatoms: I. *Cyclotella nana* Hustedt, and *Detonula confervacea* (Cleve) Gran. Can. J. Microbiol..

[40] Kim S., Park M.G. (2014). Amoebophrya Spp. from the Bloom-Forming Dinoflagellate *Cochlodinium polykrikoides*: Parasites Not Nested in the “*Amoebophrya ceratii* Complex”. J. Eukaryot. Microbiol..

[41] Adachi M., Sako Y., Ishida Y. (1994). Restriction Fragment Length Polymorphism of Ribosomal DNA Internal Transcribed Spacer and 5.8s Regions in Japanese *Alexandrium* Species (*Dinophyceae*). J. Phycol..

[42] Guillard R.R.L., Stein J.R. (1980). Handbook of Phycological Methods: Culture Methods and Growth Measurements.

