# Supplementary material for: Application of a Quantitative PCR to Investigate the Distribution and Dynamics of Two Morphologically Similar Species, Karenia mikimotoi and K. papilionacea (Dinophyceae) in Korean Coastal Waters
Source: Toxins (Basel). 2023 Jul 20;15(7):469. doi: 10.3390/toxins15070469 (PMC10467055; doi:10.3390/toxins15070469)
Supplement: Supplementary file 1 [file toxins-15-00469-s001.zip › toxins-2447547-supplementary.pdf]

Supplementary materials: Application of a Quantitative PCR to Investigate the Distribution and Dynamics of Two Morphologically Similar Species, *Karenia mikimotoi and K. papilionacea* (Dinophyceae) in Korean Coastal Waters

Sunju Kim, Minji Cho, Jiae Yoo and Bum Soo Park

Table S1. List of sampling locations, sampling dates, temperature, salinity, cell abundances (cells L^{-1}) of *K. papilionacea* (Kp) and *K. mikimotoi* (Km) estimated by qPCR assay at each site in Korean coastal waters in September 2017.

	St.	Sampling site	Latitude	Longitude	Sampling date	Temp. (°C)	Sal.	Kp	Km
East Sea	**S1**	Gangneung	37°46'19"N	128°57'04"E	3 Sep 2017	23.9	30.9	-	-
	**S2**	Daejin, Donghae	37°34'53"N	129°06'43"E	3 Sep 2017	23.9	30.9	-	-
	**S3**	Sadong, Uljin	36°49'22"N	129°26'57"E	3 Sep 2017	24.2	31.4	-	6
	**S4**	Hakri, Gijang	35°15'30"N	129°14'48"E	3 Sep 2017	24.9	30.9	1147	-
South Sea	**S5**	Yongho, Busan	35°07'57"N	129°07'15"E	8 Sep 2017	25.8	30.4	39	-
	**S6**	Jangcheon,Jinhae	35°07'40"N	128°41'55"E	3 Sep 2017	25.3	31.9	-	-
	**S7**	Dotseom, Masan	35°10'33"N	128°34'11"E	3 Sep 2017	26.3	27.7	-	-
	**S8**	Dangdong, Goseong	34°59'20"N	128°24'34"E	3 Sep 2017	27.7	31.8	-	-
	**S9**	Goseong	34°56'19"N	128°12'06"E	3 Sep 2017	26.5	33.0	-	-
	**S10**	Mijo, Namhae	34°42'53"N	128°03'06"E	3 Sep 2017	25.5	32.9	336	-
	**S11**	Bangjukpo, Yeosu	34°37'55"N	127°47'32"E	2 Sep 2017	26.0	30.0	2477	3
	**S12**	Jakgeum, Yeosu	34°35'40"N	127°45'31"E	2 Sep 2017	25.3	31.4	98	-
	**S13**	Nokdong ,Goheung	34°31'34"N	127°08'06"E	2 Sep 2017	25.3	31.4	-	-
	**S14**	Wando	34°19'38"N	126°48'32"E	2 Sep 2017	23.9	31.9	21	-
Yellow Sea	**S15**	Buk harbor, Mokpo	34°48'23"N	126°21'55"E	2 Sep 2017	24.6	28.8	-	-
	**S16**	Gyeokpo, Buan	35°37'21"N	126°28'10"E	2 Sep 2017	24.8	31.9	26	-
	**S17**	Bieung, Gunsan	35°56'18"N	126°31'52"E	2 Sep 2017	25.9	31.5	-	13
	**S18**	Magumpo, Taean	36°37'22"N	126°17'10"E	2 Sep 2017	24.4	31.5	14	122

Table S2. Calculated cell number (per mL) by direct counting and quantitative real-time PCR assay for the comparison of cell numbers between strains. qPCR values represent means ± SE.

Species	Strain	Cells number via light microscope	Cells number via standard curve	Strain for plotting standard curve
K. papilionacea	KP-LOMME02	600	639 ± 178	KP-LOMME01

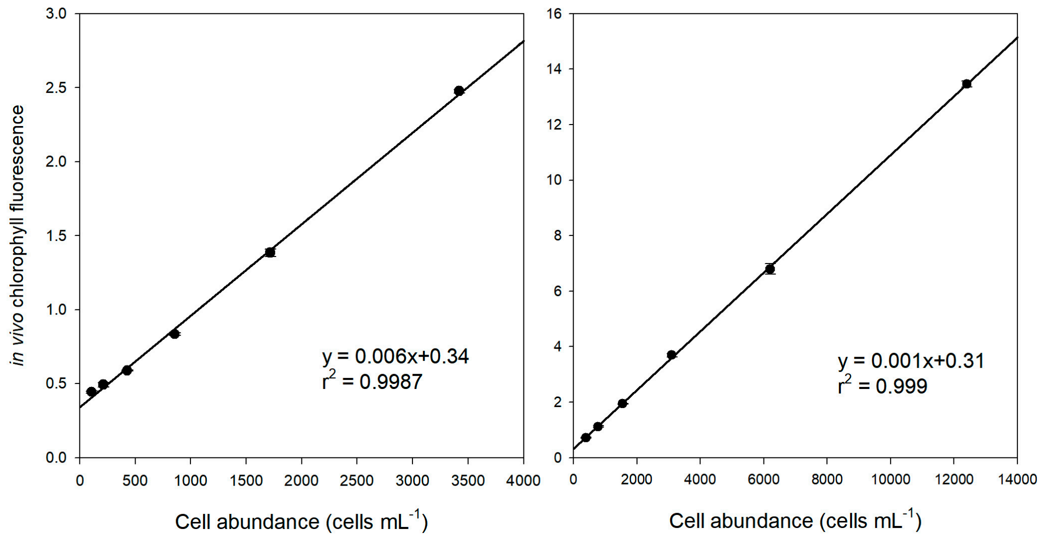

Figure S1. Relationship between cell abundance and *in vivo* chlorophyll fluorescence in *K. papilionacea* (A) and *K. mikimotoi* (B).
